# CSA: Utility Optimization Scheduling Algorithm for IoT Blockchain Sharding Committees

**DOI:** 10.3390/s25061648

**Published:** 2025-03-07

**Authors:** Xin Cong, Qi Jing, Lingling Zi, Changjiang Lin

**Affiliations:** College of Computer and Information Science, Chongqing Normal University, Chongqing 401331, China; cx@cqnu.edu.cn (X.C.); zll@cqnu.edu.cn (L.Z.); 2023210516048@stu.cqnu.edu.cn (C.L.)

**Keywords:** IoT blockchain, sharding, committee scheduling

## Abstract

The rapid proliferation of the Internet of Things (IoT) poses significant challenges for utility optimization in sharding blockchain systems. In this paper, we propose a Committee Scheduling Algorithm (CSA), which employs an iterative optimization framework based on the Markov chain to balance transaction throughput, cumulative latency, and transaction fees. CSA dynamically adjusts the committee members to achieve near-optimal solutions while addressing operational constraints. Theoretical analysis demonstrates the convergence bounds of the algorithm and its robustness against Sybil and eclipse attacks, ensuring high entropy for committee selection. Experimental results show that CSA outperforms Stochastic-Exploration (SE), Simulated Annealing (SA), and Policy Gradient-Based Computing Task Scheduling (PG-CTS) in terms of utility, convergence speed, and adaptability to dynamic events, with the committee scheduling utility improving by about 30%. Furthermore, CSA demonstrates stable performance in large-scale IoT environments characterized by dynamic node additions and failures. This paper offers a robust and adaptive solution for utility optimization in sharding blockchains, thereby improving the scalability, security, and efficiency of IoT applications.

## 1. Introduction

Blockchain technology, as a distributed ledger system, provides a revolutionary solution for decentralized transaction verification and data storage [1,2,3,4,5,6], such as dynamic sharding schemes like DBShard [7] and DRLBShard [8]. These existing solutions mainly focus on adjusting the size and number of shards to optimize system performance, which may not be suitable for resource-constrained IoT blockchain networks. Therefore, how to effectively improve the throughput and security performance of IoT blockchain sharding systems has become a key research issue.

To improve system throughput and efficiency, transactions in sharding blockchains are processed by multiple committees in parallel. However, at the beginning of each epoch, nodes take time to form committees, and the imbalance in latency between committees increases the transaction confirmation time, thereby reducing blockchain throughput. Therefore, it is necessary to investigate how to efficiently schedule committees to reduce transaction waiting time and improve system performance [9]. In addition, in dynamic IoT environments, the system faces a number of challenges that can impact the stability of committees and the overall performance of the system. For example, joining and leaving of nodes, fluctuations in transmission latency, and attacks by malicious nodes can lead to changes in committee members, which impacts the stability of the system [10]. Existing sharding protocols generally perform well in static network environments, but are relatively poorly adapted to dynamic changes, which may lead to performance fluctuations and security risks. Therefore, establishing effective scheduling committees to optimize system performance and ensure security in dynamic environments becomes a feasible way to address these challenges.

In this paper, a committee utility optimization scheduling algorithm (CSA) is proposed for IoT blockchain sharding. The CSA algorithm can dynamically adapt to the changes of the committees in real time, autonomously select the optimal committee, and quickly adjust the scheduling strategy when the committee members change. This ensures high throughput and stability in dynamic environments. In addition, CSA offers low latency, high real-time performance, and robust security against attacks such as Sybil and eclipse attacks. The proposed optimization algorithm not only solves the problem of inefficient scheduling in IoT blockchain systems, but also enhances the robustness of the system to handle dynamic node changes, network latency fluctuations, and various security challenges. Specifically, the contributions of this paper are described as follows.

- We propose an IoT blockchain Committee Scheduling Algorithm (CSA), which enables the system to prioritize committees with strong processing capabilities and low latency, thereby enhancing system efficiency and reducing transaction latency.
- CSA improves the committee scheduling optimization framework by incorporating transaction fees as a metric, making the system’s utility considerations more comprehensive and optimized. By designing a well-structured Markov chain state space and transition probability matrix, it achieves iterative optimization among feasible solutions, ultimately selecting the best committee scheduling scheme, thus addressing the inefficiency issues in IoT blockchain systems.
- CSA adopts Kullback–Leibler (KL) divergence to analyze performance disturbances, replacing the traditional total variation distance, thus providing a more precise method for measuring distribution deviations and offering a solid theoretical foundation for disturbance analysis in complex systems. The committee scheduling optimization framework effectively handles uncertainties such as dynamic node changes and network latency fluctuations in IoT environments, and can mitigate security threats like Sybil and eclipse attacks, thereby enhancing the system’s security.

The remainder of this paper is organized as follows: Section 2 reviews related work on blockchain sharding and IoT blockchain technologies. Section 3 presents the proposed CSA, including problem definition, technical details, algorithm implementation, and convergence analysis. Section 4 offers a theoretical analysis and performance evaluation. Finally, Section 5 summarizes the paper and discusses future research directions.

## 2. Related Work

Blockchain sharding technology, with its characteristics of scalability, decentralization, security, and anonymity, makes it a promising solution to address the challenges in the Internet of Things (IoT). Many representative sharding blockchain protocols have been proposed [11,12,13]. For example, Zamani et al. [14] proposed RapidChain, a fault-tolerant sharding protocol for permissionless blockchains. RapidChain leverages efficient cross-shard verification methods to improve throughput, and eliminates the need for flood broadcasting messages. To improve the efficiency of cross-shard transactions, Amiri et al. [15] introduced SharPer, a permissioned blockchain system that improves scalability by reallocating different data shards to different network clusters. Wang et al. [16] proposed a blockchain sharding scheme—Node Rating Sharding (NRS). This approach evaluates and selects nodes with high reputation for shard participation, thereby improving system security and shard resilience while reducing threats from nodes with low reputation. Nguyen et al. [17] introduced OptChain, a new shard placement approach aimed at reducing the number of cross-shard transactions. Similarly, Dang et al. [18] developed a generic distributed transaction protocol for blockchain sharding, which improves the efficiency of shard generation by utilizing Elastico.

Regarding IoT blockchain systems, scholars have recently proposed several methods and projects. For example, Aptos adopted a new consensus protocol that optimizes the throughput and scalability of a sharding network, with a focus on improving cross-chain performance and security [19]. Sui used the Narwhal consensus mechanism to improve system throughput and parallel processing of transactions, especially for IoT applications with low-latency requirements [20]. Linera employed a hybrid protocol combining asynchronous and synchronous consensus that optimizes the integration of sharding and blockchain and improves system efficiency [21]. Fuel utilized parallel execution technology to reduce transaction latency and enhance throughput, making it an ideal solution for IoT networks with high-performance requirements [22]. Wang et al. [23] proposed TransShard, a transaction-aware sharding scheme that dynamically adjusts the sharding strategy according to the transaction characteristics of account-based blockchains, thus improving the transaction efficiency and system throughput. Wei et al. [24] presented FCDSB, a fog computing network architecture based on dynamic blockchain sharding.FCDSB is designed for consumer electronics in the Artificial Intelligence of Things (AIoT) to optimize the allocation of computing resources and enhance security. Li et al. [25] proposed a dynamic blockchain sharding scheme based on graph partitioning, and they optimized load balancing and cross-shard communication efficiency using the graph partitioning methods. Regarding the storage issue, Peng et al. [26] presented a clustering-based collaborative storage scheme for blockchain applications in IoT systems. This scheme optimizes storage allocation among nodes and improves the storage efficiency and scalability of the blockchain network. Additionally, Li et al. [27] proposed a secure and efficient blockchain sharding scheme by combining hybrid consensus mechanisms and dynamic management, which effectively enhances the security and fault tolerance of the system. Furthermore, Hu et al. [28] introduced LMChain, an efficient load-migratable beacon-based sharding blockchain system, which further improves the scalability and efficiency of IoT blockchain systems through dynamic load migration and beacon-based sharding mechanisms.

In IoT environments, scholars have proposed an innovative dynamic and adaptive sharding framework, called SmartChain [29], specifically designed to overcome the inherent challenges of dynamism and heterogeneity in IoT blockchain systems. SmartChain dynamically selects the number of shards, partition structures, and leader selection modes to achieve high throughput and low security risks. To address the problem of limited scalability of IoT frameworks, Wu et al. [30] proposed a novel blockchain-integrated sharding algorithm by analysing transaction records of frequently interacting sender–receiver pairs. Furthermore, considering the uniqueness of IoT environments, Xi et al. [31] proposed HMMDShard, a dynamic blockchain sharding scheme based on hidden Markov models (HMMs). HMMDShard achieves adaptive dynamic incremental updates of blockchain shards by integrating HMMs, effectively reducing cross-shard transactions across all shards. Aiming at storage and computation issues in IoT environments, Pan et al. [32] proposed HyperChain, a dynamic state sharding protocol that supports smart contract execution, which achieves low cross-shard communication and good scalability. This scheme optimizes the efficiency of cross-shard communication through intelligent sharding strategies, enhancing the overall performance of the blockchain. Based on the above analysis, most solutions focus on shard generation, optimization of cross-shard transactions, and improving system throughput and security. However, there is still room for further improvements in enhancing overall system efficiency, addressing challenges in dynamic IoT environments, and improving cross-shard communication efficiency.

In summary, we have summarized typical related works in Table 1. To address the issue of unbalanced two-phase latency caused by member committees in IoT environments and to accelerate the process of performing final consensus in the final committee, we propose our algorithm, CSA. This algorithm aims to improve block generation efficiency and the overall throughput of the blockchain system while enhancing system security.

## 3. CSA Algorithm

In this study, we formalize the trade-off relationships among transaction throughput, cumulative transaction waiting time, and transaction fees into a utility maximization problem and propose a committee scheduling algorithm to solve it. This section introduces the proposed method and provides an analysis from the perspectives of problem definition, Markov chain design, and the CSA.

### 3.1. Algorithm Framework

**Figure 1:**
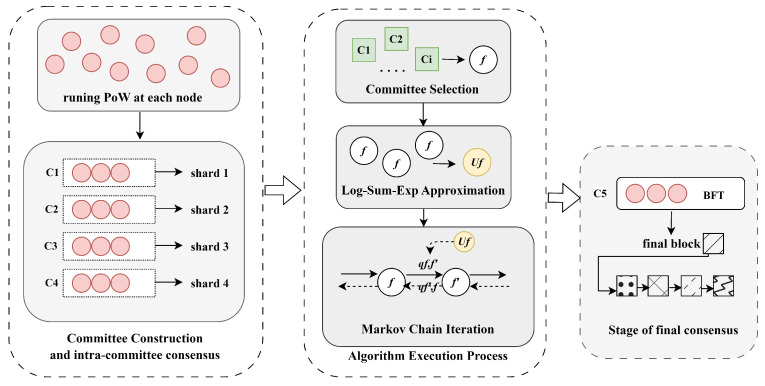
The framework of the algorithm.

The algorithm framework is shown in Figure 1. The first part is the construction of committees before the algorithm implementation and the two-stage latency of the committees. In this part, committees are formed through a proof-of-work-based election mechanism, which causes the committee formation latency. This is followed by the internal consensus phase within the committee, known as consensus latency. Then, committees are selected as feasible solutions. To make the problem clearer and solvable, we use Log-Sum-Exp approximation (see Section 3.2) to simplify the problem. Next, using the iterative optimization idea based on the Markov chain (see Section 3.3), feasible solutions are continuously iterated and optimized to select the optimal solution based on the transition probability matrix. The specific process of the algorithm (see Section 3.4) includes the algorithm flow and the derivation of the algorithm’s convergence. Finally, the best committee combination selected is submitted to the final committee, forming the final block.

The committee scheduling problem is modeled in a blockchain sharding system, which operates within epochs J={1,2,…,j,…,}. The committees are divided into member committees and the final committee. The final committee is responsible for generating the final block on the root chain, while the other committees are referred to as member committees. The shards generated by the member committees are represented by the set $I^{j}=\left\{1,2,3,\ldots ,i,\ldots \right\}$. A binary variable $x_{i}^{j}\in \left\{0,1\right\}$ is defined to indicate whether committee $i\in I^{j}$ is allowed to participate in the final consensus phase of period j∈J. The total two-phase latency set of each member committee is represented by $L^{j}=\left\{l_{i}^{j},i\in I^{j}\right\}$, while the number of transactions in each committee is denoted by $S^{j}=\left\{s_{i}^{j},i\in I^{j}\right\}$. Additionally, $t^{j}$ is introduced to represent the given deadline (DDL) used to evaluate the shards received in epoch *j*.

When the transaction capacity of the final block is constrained, a predefined DDL should be minimized to expedite the generation process of the final block. In the experiments, the DDL is defined as a fixed proportion of the time taken by the member committees to submit their shards to the designated committee. Consequently, the DDL is defined as $t^{j}=\max_{i\in I^{j}}l_{i}^{j},\;\forall j\in J$, which can be calculated as the sum of the time from the committee formation phase to the specified DDL. Furthermore, the cumulative time $\Pi_{i}^{j}$ for all transactions encapsulated in each shard $i\in I^{j}$ can be computed.

(1) $$\Pi_{i}^{j}=x_{i}^{j}\left(t^{j}-l_{i}^{j}\right)=x_{i}^{j}(\max_{k\in I^{j}}l_{k}^{j}-l_{i}^{j}),\;\forall i\in I^{j},\;j\in J$$

The objectives of the problem are to (1) maximize the total number of transactions processed by the final committee, (2) reduce the overall cumulative time for these transactions, and (3) increase the transaction fees collected by the committees. The first objective aligns with improving the throughput of the main chain, the second relates to enhancing the timeliness of transactions distributed across allowed shards, and the third focuses on optimizing the committees’ efficiency and boosting the blockchain network’s appeal. As a result, the following utility maximization problem is established:

(2) $$\mathrm{MUCom}\text{:}\;\max U=\sum_{j\in J}\sum_{i\in I_{j}}\left(\alpha \cdot x_{i}^{j}\cdot s_{i}^{j}+\lambda \cdot x_{i}^{j}\cdot A_{i}^{j}-\Pi_{i}^{j}\right)$$

The constraints are as follows:

(3) $$\sum_{i\in L_{j}}x_{i}^{j}\geq N_{\min},\;\forall j\in J$$

(4) $$\sum_{i\in L_{j}}x_{i}^{j}s_{i}^{j}\leq \hat{C},\;\forall j\in J$$

(5) $$x_{i}^{j}\in \left\{0,1\right\},\;\forall i\in I^{j},\forall j\in J$$

In the objective function MUCom, α and λ are adjustable weights that measure the preference for the total number of allowed transactions (TXs) and transaction fees. Constraint (3) specifies that the number of selected member committees in each epoch must be greater than the predefined minimum $N_{\min}$. In constraint (4), $\hat{C}$ represents the capacity of transactions that can be packaged into the final block during each epoch. Therefore, constraint (4) ensures that the capacity limit of the final block is maintained in every epoch j∈J.

### 3.2. Log-Sum-Exp Approximation

**Figure 2:**
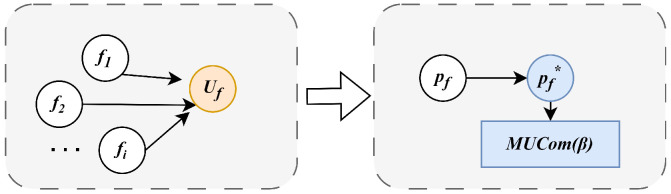
The approximation process of the problem.

Using the log-sum-exp approximation, the optimal solution to the original problem is represented as a probability distribution, enabling the algorithm to probabilistically identify near-optimal solutions. As shown in Figure 2, let *f* denote a feasible solution to the target problem, where each feasible solution is associated with a probability $p_{f}$, indicating the likelihood of the system selecting *f*. This approach defines the problem, approximated by the log-sum-exp technique, as the MUCom(β) problem [33], where β∈(0,∞) is a positive scalar that governs the approximation accuracy. The set of approximations of all feasible solutions is regarded as the state space of the Markov chain, and the design of an effective transition probability matrix helps to obtain the optimal solution.

### 3.3. Markov Chain Design

To address the iterative transitions between different committee state sets, a Markov chain-based state transition algorithm is used to optimise the scheduling problem of committees in a sharded blockchain system. Specifically, at each time interval, the system selects the next state according to state transition rules, forming a Markov chain that converges to the optimal solution. In the model, the scheduling scheme for each committee is represented as a state *f*, and the entire state space consists of all possible combinations of committee schedules. For each pair of adjacent states $f,f^{\prime }\in F$, a transition probability $q_{f,f^{\prime }}$ is defined, which is linked to the utility difference between the two states. In the Markov chain, all states are mutually reachable, and for every pair of adjacent states $f,f^{\prime }\in F$, the detailed balance equation $p_{f}q_{f,f^{\prime }}=p_{f^{\prime }}q_{f^{\prime },f}$ [34,35] must hold.

**Figure 3:**
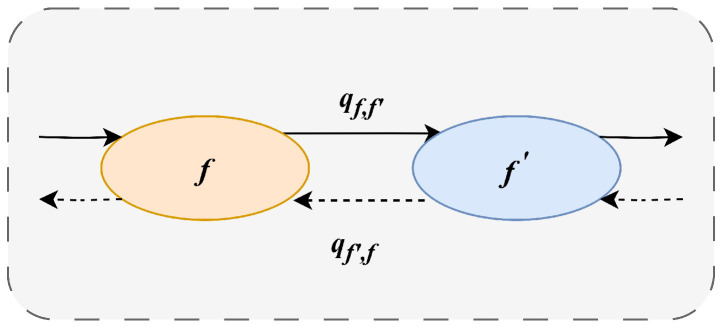
Transition between neighboring states.

Initially, the transition rate between two solutions *f* and $f^{\prime }$ is set to zero until they simultaneously satisfy the following two conditions: (a): $\left|f\cup f^{\prime }\right|-\left|f\cap f^{\prime }\right|=2$. (b): $f\cup f^{\prime }-f\cap f^{\prime }\in x_{i}$, where *i* represents the shard responsible for the transition $f\to f^{\prime }$. In other words, transitions between states do not occur until specific conditions are met, after which state transitions are allowed. This controlled transition ensures local changes in the system rather than large-scale adjustments, which will help the system to remain stable and gradually converge to the optimal solution in the dynamically changing environment of large-scale IoT nodes. The state transitions are shown in Figure 3. The state transition probabilities for the required Markov chain are designed as follows:

(6) $$q_{f,f^{\prime }}=\frac{1}{\exp\left(\tau \right)}\exp\left(\frac{1}{2}\beta \left(U_{f^{\prime }}-U_{f}\right)\right),\;\forall f,f^{\prime }\in F$$

With this design, the system gradually reaches high-utility states, achieving higher efficiency in committee scheduling and transaction verification, while reducing latency and optimizing overall performance. The transition probabilities are designed to gradually approach the optimal state, thereby reducing the latency in committee scheduling and mitigating performance perturbations within the system. Let $p_{f}^{*}\left(f\in F\right)$ represent the optimal solution to the MUCom(β) problem. By solving the Karush–Kuhn–Tucker (KKT) conditions [36] for MUCom(β), the steady-state probability for each solution f∈F can be derived as follows:

(7) $$p_{f}^{*}=\frac{\exp\left(\beta U_{f}\right)}{\sum_{f^{\prime }\in F}\exp\left(\beta U_{f^{\prime }}\right)},\;\forall f\in F$$

Due to the iterative nature of the system, state transitions in the designed Markov space correspond to the replacement of member committee sets. Therefore, during the implementation of Markov chain, if the state transitions are trained to converge to the desired steady-state distribution $p_{f}^{*}$, the system will achieve near-optimal performance. This Markov chain is designed to deviate from time reversibility. Such an approximation algorithm can converge to the globally optimal state relatively quickly. In addition, by adjusting the design of the state transition probabilities, the algorithm ensures that the system maintains robust performance in the presence of dynamic events such as committee members joining or leaving, thus realizing adaptive scheduling adjustments.

Theoretically, the designed Markov chain has good convergence and its mixing time $t_{\mathrm{mix}}\left(\epsilon \right)$ [35] is proportional to the complexity of the system’s states, as shown below.

(8) $$t_{\mathrm{mix}}\left(\epsilon \right)=\inf\left\{t\geq 0:\max D_{\mathrm{KL}}\left(H_{t}\left(f\right)∥p^{*}\right)\leq \epsilon \right\}$$

Meanwhile, by adjusting the parameters of the transition probabilities, the algorithm can effectively control the system’s convergence speed and its adaptability in dynamic environments. Experimental results also show that the Markov chain algorithm exhibits strong adaptability and optimization performance in solving complex committee scheduling problems.

### 3.4. CSA: Committee Utility Optimization Scheduling Algorithm

This section first describes the implementation of the CSA, including the solution construction, algorithm iteration, and real-time monitoring process. Then, the convergence and complexity of the algorithm are discussed.

#### 3.4.1. Algorithm Implementation

An implementation of the proposed CSA based on Markov chain theory with a well-designed transition-rate matrix is presented in Algorithm 1. CSA is designed to schedule blockchain committees to achieve the optimal solution *f*. In Lines 1 through 2, it first checks whether the number of committees exceeds the minimum threshold $N_{\min}$ and whether the total number of transactions received exceeds the capacity *C*. If these conditions are satisfied, the event monitoring process is initiated for the current epoch. Then, in Lines 3 through 7, the algorithm generates possible committee combinations by iteratively constructing the initial solution set $f_{n}$ while ensuring that the constraints are satisfied.

**Figure 4:**
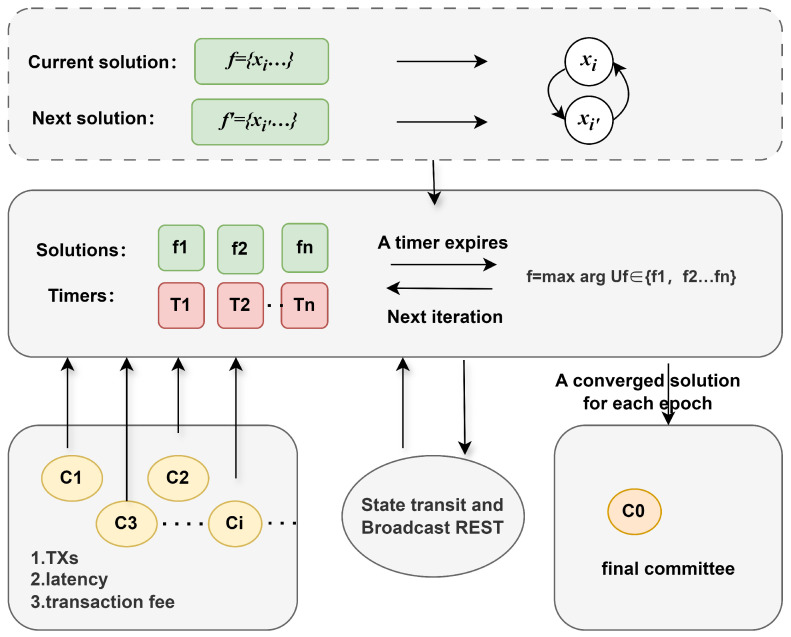
Algorithm execution process.

During each iteration, the algorithm randomly selects two committees *i* and $i^{\prime }$ from the current state, setting $x_{i}^{n}=1$ and $x_{i^{\prime }}^{n}=0$, respectively. It calculates the current system utility $U_{f}$ and the hypothetical utility $U_{f}^{\prime }$, then uses these values to generate a timer $T_{n}$ for continuous monitoring. Figure 4 shows the execution process of the algorithm, where the algorithm runs on multiple independent parallel threads. Each thread executes a set of feasible solutions $\left\{f_{n}\right\}\left(n=1,2,\ldots ,\right|I^{j}\left|-1\right)$ and timers $\left\{T_{n}\right\}\left(n=1,2,\ldots ,\right|I^{j}\left|-1\right)$. All parallel threads communicate with the committees in real time, exchanging a limited set of messages such as reset signals and the current system utility.

When a committee joins or leaves, the algorithm dynamically updates the committee set $I^{j}$, resets the initial solution set $f_{n}$, and reinitialises the timer $T_{n}$. When a timer $T_{n}$ expires, the algorithm exchanges the corresponding committee states and sends a RESET signal to notify other nodes to recalibrate. In addition, the algorithm supports responding to RESET signals by recalculating the timer and adjusting the states accordingly.

The algorithm terminates under two conditions: (1) when the system states converge and the system satisfies the capacity constraint *C*, the solution with the highest utility is chosen as the final solution *f*; (2) when the number of final committees exceeds the maximum threshold $N_{\max}$, the algorithm stops monitoring new incoming committees. Through this process, the algorithm achieves optimal committee scheduling even in dynamic environments.
**Algorithm 1:** Committee Scheduling Algorithm
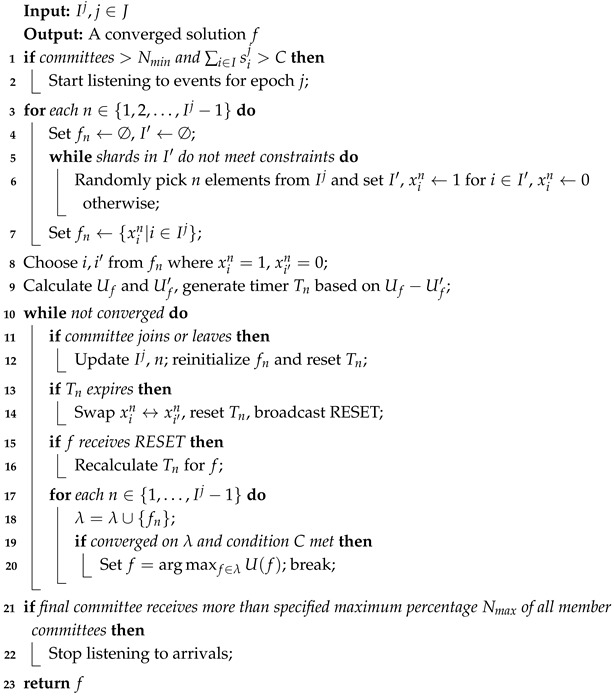


#### 3.4.2. Algorithm Convergence

The time required for the Markov chain to reach convergence can be quantified using the concept of mixing time [35]. Let $H_{t}\left(f\right)$ be the probability distribution over all states in the solution space F at time *t*, where the initial state is *f*, and let $p^{*}$ be the static distribution probability of the designed Markov chain. The mixing time can then be defined using the Kullback–Leibler (KL) divergence as follows:

(9) $$t_{\mathrm{mix}}\left(\epsilon \right)=\inf\left\{t\geq 0:\max D_{\mathrm{KL}}\left(H_{t}\left(f\right)∥p^{*}\right)\leq \epsilon \right\}$$

In this context, ϵ>0 denotes the allowable deviation between the converged solution and the optimal solution, while $D_{\mathrm{KL}}$ represents the KL divergence, which quantifies the discrepancy between two probability distributions. For each epoch j∈J, the cardinality of the feasible solution space can be expressed as follows:

(10) η=∑n=0|Ij||Ij|n=2|Ij|

The maximum number of transition options is |Nfn|=∑n=1|Ij|−1(|Ij|1)(|Ij|−1n)=12(|Ij|2−|Ij|). The iterative convergence of the proposed algorithm leads to the following conclusions: For a given set of committees, let $U_{\max}=\max U_{f}$ and $U_{\min}=\min U_{f}$. With ϵ>0, the Markov chain has the following constraints on the mixing time $t_{\mathrm{mix}}\left(\epsilon \right)$ in each epoch [35,37]:

(11) $$t_{\mathrm{mix}}\left(\epsilon \right)\geq \frac{\exp\left[-\frac{1}{2}\beta \left(U_{\max}-U_{\min}\right)\right]}{|I^{j}{|}^{2}-\left|I^{j}\right|}\ln\frac{1}{2\epsilon }$$

(12) $$\begin{array}{cc} t_{\mathrm{mix}}\left(\epsilon \right) & \leq 4^{|I^{j}|}\left(\right|I^{j}{|}^{2}-|I^{j}\left|\right)\exp\left[\frac{3}{2}\beta \left(U_{\max}-U_{\min}\right)+\tau \right]\\ \; & \times \left[\ln\frac{1}{2\epsilon }+\frac{1}{2}\left|I_{j}\right|\ln2\right]+\frac{1}{2}\beta \left(U_{\max}-U_{\min}\right) \end{array}$$

As $|I^{j}|$ increases, the convergence time $t_{\mathrm{mix}}\left(\epsilon \right)$ has an upper bound. It also grows exponentially with exp(β) and logarithmically with $\ln\frac{1}{\epsilon }$. Thus, the pursuit of smaller performance loss (i.e., smaller ϵ) leads to a higher upper bound on the convergence time and vice versa. Additionally, as the number of transactions increases, the algorithm needs to process more transactions and dynamic events, which increases the computational overhead. The convergence time also increases as the solution space becomes more complex, with state transitions growing more complex as $|I^{j}|$ grows. Therefore, while the algorithm performs well in the simulation experiment, the convergence time still increases significantly as the scale grows.

Next, we analyze the computational complexity of the algorithm. The maximum number of transition options between states is $O(|I^{j}{|}^{2})$. Although the computation of each individual transition probability is O(1), the overall complexity is $O(|I^{j}{|}^{4})$, since the transition space is $O(|I^{j}{|}^{2})$, and each state can transition with other $O(|I^{j}{|}^{2})$ states. The space complexity is primarily determined by the space required to store the state space F and the transition probability matrix. Since the size of the state space is $O(n^{2})$, the space required to store the state and transition matrix is $O(n^{4})$. We further explore the trade-off between the optimal solution loss (expressed as $\frac{1}{2}\beta \ln\left|F\right|$), and the mixing time for different values of β. As β approaches infinity, the optimal solution loss decreases asymptotically to zero. However, the upper bound of the mixing time grows exponentially at the rate of O(exp(β)) and diverges to infinity. Consequently, while a larger β is effective in minimizing the loss of the solution, it greatly affects the convergence speed, highlighting the important balance between solution accuracy and computational efficiency.

## 4. Theoretical Analysis and Experimental Simulations

In order to comprehensively evaluate the performance of the proposed algorithm in the IoT environment, we first provide a theoretical analysis of Sybil attacks, eclipse attacks, and committee performance perturbations to demonstrate the reliability and accuracy of the algorithm. Then, we perform experiments to evaluate the performance of the algorithm.

### 4.1. Theoretical Analysis

In the IoT environment, blockchain sharding systems are more vulnerable to malicious attacks. On the one hand, the large number of device nodes in IoT environments often exhibit resource inequality, and this imbalance makes the IoT system more susceptible to malicious attacks such as Sybil attacks. On the other hand, IoT networks are highly dynamic and heterogeneous, and the committee members in the network are difficult to predict and control, which provides more opportunities for eclipse attacks and DDoS (Distributed Denial of Service) attacks. Therefore, we analyze the failures of these two types of attacks and investigate the extent of committee performance disruptions.

#### 4.1.1. Analysis of Sybil Attacks

**Figure 5:**
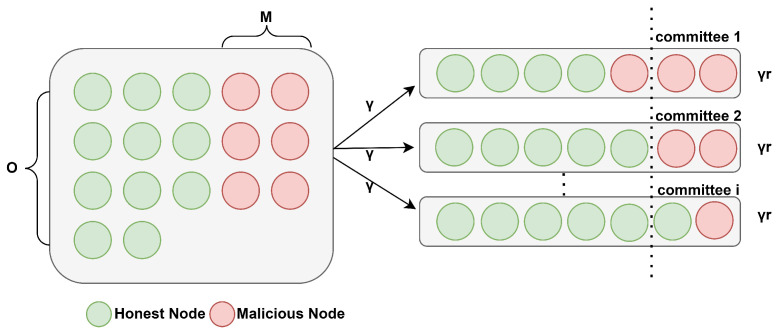
The attack model of the algorithm.

During the committee formation phase, malicious Sybil nodes might be allocated to a committee. If their proportion exceeds the tolerance threshold (e.g., $\frac{1}{3}$ for PBFT protocol), the committee will fail. In this paper, we consider an attack model where the blockchain network comprises comprises *O* nodes, with *M* nodes (M<O) being malicious nodes, as shown in Figure 5. When the *O* nodes are distributed in committees with a number of $|I^{j}|$, each committee contains $\gamma =\left\lfloor \frac{O}{|I^{j}|}\right\rfloor$ nodes on average. That is, each committee contains $\gamma =\left\lfloor \frac{O}{|I^{j}|}\right\rfloor$ nodes, comprising both malicious and honest nodes. Let r∈(0,1] represent the resilience of a member committee, indicating the maximum proportion of malicious nodes it can withstand. For example, in the PBFT protocol, $r=\frac{1}{3}$, and the likelihood of a single-member committee failing due to a Sybil attack is expressed as follows [9]:

(13) P1=|Ij|×∑mw=⌊γr⌋+1min{γ,M}MmwO−Mγ−mw/Oγ×Ψ(M−mw)(0)(M−mw)!O−γM−mw

The probability of multiple committees failing can be expressed as

(14) P3=1−P1−Ψ2(M)(0)M!OM,

#### 4.1.2. Analysis of Eclipse Attacks

An eclipse attack is a network-based attack in which an attacker creates an environment to manipulate miners into taking incorrect actions. This type of attack relies on the entropy value (randomness) of the system. If the entropy value is high, it is more difficult to predict the next committee member, thus invalidating the attack. The entropy value in the system is defined as follows:

(15) $$H\left(X\right)=-\sum_{i=1}^{n}P\left(u_{i}\right)\log_{b}P\left(u_{i}\right)$$

In this context, $P(u_{i})$ represents the probability that committee $u_{i}$ will participate in the final consensus, and *b* denotes the base of the logarithm. The election probability of a committee is proportional to its resource value. Experimental results show that the proposed algorithm achieves a higher entropy value. High entropy implies a higher level of security, as it increases the unpredictability of the next committee members.

This is achieved by randomly initializing the committees in each epoch and then selecting it according to the Markov iterative optimization principle. In addition, rejected committees are treated appropriately in subsequent epochs. Specifically, rejected committees refresh the DDL in the next epoch, which reduces their two-stage latency and makes them more likely to be selected. This mechanism makes it difficult for an attacker to predict the next committee member, thus effectively preventing targeted attacks.

#### 4.1.3. Analysis of DDoS Attacks

Distributed Denial of Service (DDoS) attacks can cause disruption of normal service by sending a large number of malicious requests to target nodes, consuming their computing resources or bandwidth. In a blockchain environment, DDoS attacks may target miners or nodes in an attempt to send a large number of meaningless requests to nodes in the blockchain network, overloading them and thus preventing them from processing normal transactions or blocks.

The proposed CSA algorithm employs high entropy to randomly select member committees, which means that the committee selection in each epoch is highly randomized. Through the iterative optimization of the algorithm, the system can gradually adjust and optimize the selection of member committees in each epoch, improving the adaptability of the system when facing external attacks. This makes it difficult for DDoS attackers to predict the next set of committee members, thus reducing the probability of a successful attack. Moreover, even if attackers use large-scale traffic attacks against certain nodes, they cannot control or influence the consensus process of the blockchain network by predicting committee members. The CSA algorithm has a recovery mechanism so that if a committee temporarily fails due to a DDoS attack, the system can quickly resume normal operation by adjusting and reselecting members. If a large number of nodes fail, the system can also quickly adjust and even resume normal operation through recovery mechanisms, ensuring the stability of the blockchain network.

#### 4.1.4. Committee Performance Deviation

When a committee fails, the feasible solutions associated with that committee become invalid. As a result, these solutions need to be eliminated from the original solution space F, which passes into the pruned space G. The set of solutions removed from F is stored as F∖G. Specifically, if a single committee fails, the optimal feasible solution [i,j] is removed first, and then all candidate solutions containing the failed committee (denoted as $F_{n}\;(n=1,2,\cdots ,|I^{j}\left|-1\right)$) are migrated into F∖G.

Furthermore, the corresponding transition rates associated with these solutions also become invalid. It can be shown that the newly truncated Markov chain remains unchanged even if the invalid solutions and their corresponding transition probabilities are removed. Therefore, the CSA only needs to maintain the pruned solution space and the updated transition probability matrix in real time.

Next, we analyze the performance perturbation caused by a given number of committee failures. Starting from an initial state where all member committees are online, let $K\in Z,\;(1\leq K\leq |I^{j}|-1)$ represent the number of failed committees. In the truncated Markov chain, the static distribution of solutions is expressed as $q^{*}\left(u\right),u\in G$. We define another vector $q=[q_{g}],g\in G$ to represent the distribution when *K* committees fail. To quantify the difference between these two distributions, the Kullback–Leibler divergence (KL divergence) is used instead of the total variation distance. Kullback–Leibler (KL) divergence is an asymmetric measure of the difference between two probability distributions. Specifically, the KL divergence $D_{\mathrm{KL}}\left(P\|Q\right)$ quantifies the information loss or additional “information” introduced when approximating distribution *P* using distribution *Q*. Its formula is given by

(16) $$D_{\mathrm{KL}}\left(q^{*}∥q\right)=\sum_{g\in G}q_{g}^{*}\log\frac{q_{g}^{*}}{q_{g}}$$

When κ$\left(1\leq \kappa \leq |I^{j}|-1\right)$ committees fail, the KL divergence between $q^{*}$ and $\tilde{q}$ is expressed as

(17) $$D_{\mathrm{KL}}\left(q^{*}∥\tilde{q}\right)\leq 2{\left(1-\frac{1}{2^{\kappa }}\right)}^{2}.$$

Additionally, when κ$\left(1\leq \kappa \leq |I^{j}|-1\right)$ committees fail, the runtime performance perturbation is described as

(18) $$∥q^{*}u^{T}-\tilde{q}u^{T}∥\leq \left(2-\frac{1}{2^{\kappa -1}}\right)\max_{g\in G}U_{g},$$

Although committee failures may reduce the utility to $\left(2-\frac{1}{2^{\kappa -1}}\right)\max_{g\in G}U_{g}$, the proposed algorithm still functions properly. When only one committee fails, i.e., K=1, the maximum performance degradation is $\max_{g\in G}U_{g}$. In the extreme scenario where $K=|I^{j}|-1$, the upper bound on the power dissipation is $\left(2-\frac{1}{2^{|I^{j}|-2}}\right)\max_{g\in G}U_{g}$. As $|I^{j}|\to \infty$, this upper bound is constrained to $2\max_{g\in G}U_{g}$. In other words, as the number of network nodes in an IoT environment increases, the performance perturbation of the algorithm is controlled within a certain range.

In other words, as the number of network nodes in an IoT environment increases, the performance perturbation of the algorithm is controlled within a certain range. The impact of its committee scheduling algorithm on system performance can be efficiently assessed by converting simple probability differences into information entropy differences using KL divergence.

### 4.2. Experimental Simulation

In our experiments, we utilized the Block Transaction dataset [38], which is a random snapshot of Ethereum historical transaction data. The transaction data include attributes such as blockID, bhash (block hash), btime (block creation timestamp), value (transaction amount), and txs (number of transactions). During each epoch, these transactions were partitioned into distinct groups to simulate transaction shards created by member committees. Meanwhile, We built a small-scale IoT system using Softether VPN 4.42 Build 9798 RTM. The latency range was set between 10 and 200 milliseconds and some high-latency, low-bandwidth nodes were configured to simulate the real environment of the IoT system. To simulate a large-scale IoT environment, the number of committees was set to 20, consisting of 19 member committees and 1 final committee. The total transaction weight α ranged from 0.5 to 2, while the transaction fee weight λ ranged from 0.1 to 1. The parameters β and τ were set to 1.5 and 0. During the execution of the algorithm, $N_{\min}$ and $N_{\max}$ were set to 50% and 80% of $|I^{j}|$, respectively. To take into account the latency between member committees, the experiments computed two-phase latency for each shard. The consensus latency for each committee was determined by executing the PBFT protocol, while the overall consensus latency was calculated based on the weighted average of the time spent in its three phases.

#### 4.2.1. Experimental Results

In the experiments, the proposed algorithm is compared with other scheduling algorithms, and the evaluation metric uses the utility value. The utility value is positively correlated with the number of transactions and transaction fees, and negatively correlated with the cumulative transaction waiting time. In other words, the higher the utility value, the better the performance.

**Figure 6:**
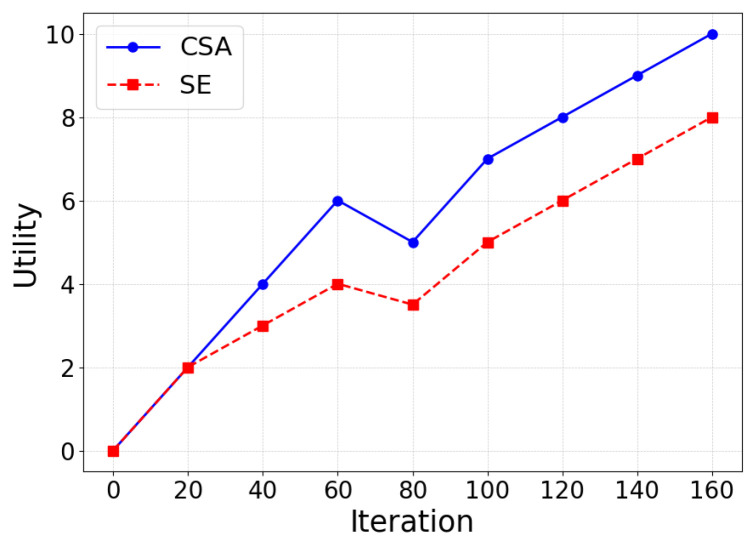
Comparison of algorithm iterations between CSA and SE.

First, the committee scheduling algorithm (SE) and our CSA are compared with a smaller number of iterations. The SE algorithm determines the optimal committee set of the blockchain through iterative optimization, which is one of the best performing algorithms in the field of committee scheduling. As shown in Figure 6, it can be seen that during this iteration, the CSA iterative process is generally similar to that of the SE. However, due to the inclusion of the transaction fee metric in the CSA, the utility of the committees demonstrated by the proposed algorithm is slightly higher. Additionally, CSA is able to converge quickly to the maximum value in a shorter time, reflecting better scheduling performance.

**Figure 7:**
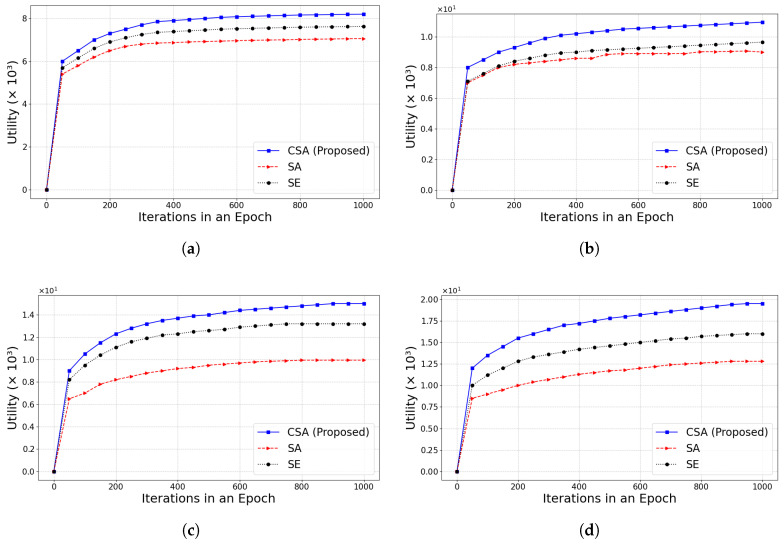
The convergence of the algorithm with $|I^{j}|$ = 25 and λ = 0.1, while changing α∈{0.5,1,1.5,2}. (**a**) α=0.5; (**b**) α=1; (**c**) α=1.5; (**d**) α=2.

Next, the CSA is compared with the SE and simulated annealing optimization algorithm (SA) when the number of iterations is larger. SA [39] is a classical heuristic algorithm that mimics the metal annealing process to search for the global optimal solution. As shown in Figure 7, the experimental results are demonstrated for different values of α under the condition of λ = 0.1. The α value represents the impact of the transaction number weight on the utility value. The utility value increases rapidly at the beginning of the algorithm execution and converges to a higher level after about 100 iterations. As the iterations proceed, the utility value of the system gradually converges to the optimal level. The experimental results show that the utility value gradually increases as α increases. Once α exceeds 0.5, the gap in utility between the scheduling algorithm and other optimization algorithms gradually widens, but the growth rate becomes slower. CSA has obvious advantages in both convergence speed and final utility.

**Figure 8:**
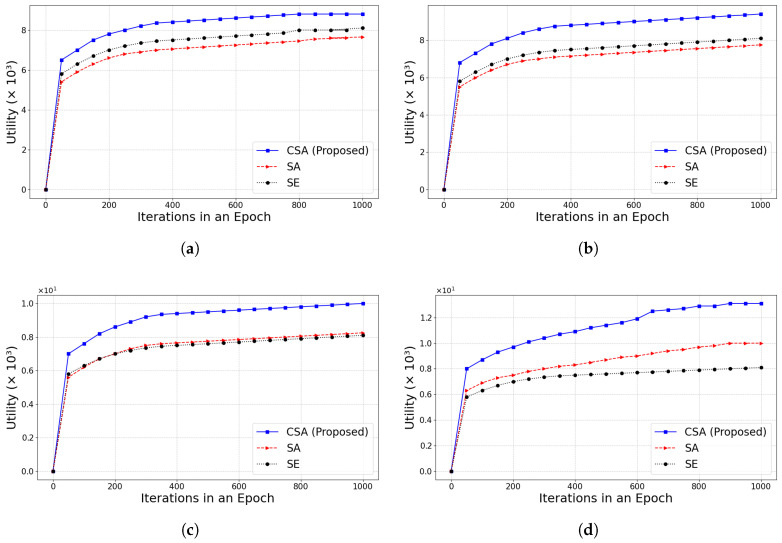
The convergence of the algorithm with $|I^{j}|$ = 25 and α = 0.5, while changing λ∈{0.1,0.3,0.5,1}. (**a**) λ=0.1; (**b**) λ=0.3; (**c**) λ=0.5; (**d**) λ=1.

We conducted experiments to compare the impact of different λ under the same conditions. As illustrated in Figure 8, as λ increased, the impact of transaction fees on total utility becomes more pronounced as λ increases. Under different λ, the utility of CSA consistently outperformed both SA and SE. Moreover, the gap of utility between CSA and the other algorithms continued to widen as λ increased. The analysis shows that the convergence to the optimal value is fastest when α = 0.5 and λ = 0.1. The experimental results showed that the CSA algorithm is better at balancing transaction fees with other optimization objectives (e.g., transaction number and latency), making it more adaptable to different weighting parameters and exhibiting better performance in IoT environments.

**Figure 9:**
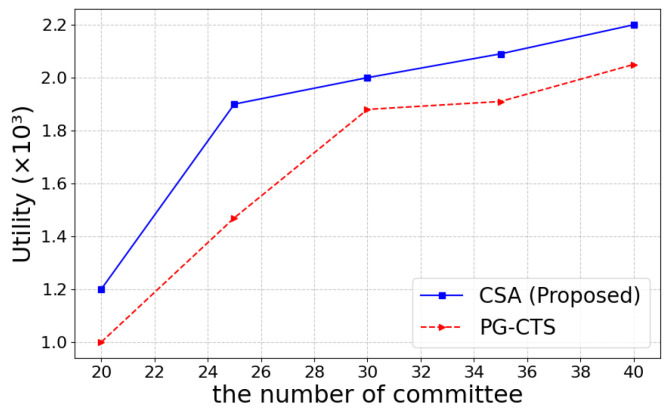
Utility comparison between CSA and PG-CTS.

In addition, we compare CSA to the Policy Gradient Computing Task Scheduling Optimization Algorithm (PG-CTS) [39] based on deep reinforcement learning. Figure 9 shows the final utility values of the two algorithms for different committee number. The comparison results indicate that both the proposed algorithm and PG-CTS show an increasing trend as the committee number increases. When the committee number exceeds 25, the gap between the two gradually widens, with the CSA algorithm showing more significant advantages. Therefore, it can be concluded that CSA has better scalability in large-scale systems compared to the PG-CTS.

#### 4.2.2. Dynamic Event Handling

**Figure 10:**
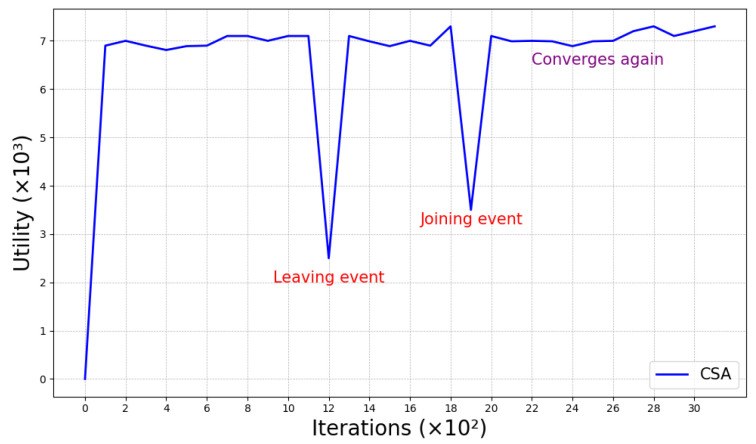
CSA performance with dynamic events.

To simulate dynamic events in the IoT environment, a dynamic event simulation function was included in the experiments. This function manually forced the addition and removal of specific committees to simulate dynamic events such as committee failures due to network anomalies (i.e., removal events) and their subsequent recovery within a short period of time (i.e., addition events). As illustrated in Figure 10, the experimental results show that the proposed algorithm is able to recover quickly within a short period of time. This proves that the scheduling algorithm can effectively handle dynamic events involving committee leaving and joining.

#### 4.2.3. Discussion

The algorithm proposed in this paper, CSA, shows good performance in experiments. First, in committee scheduling, CSA outperforms SE and SA algorithms, showing higher utility values and faster convergence. Particularly, under different values of α and λ, CSA is able to better balance the optimization objectives such as transaction fees, transaction volume, and latency, and adapt to varying weight parameters. As the values of α and λ increase, the performance gap between the CSA and other algorithms continues to widen.

Additionally, the experiments verify the scalability of the CSA in large-scale systems, which outperforms the deep reinforcement learning-based scheduling algorithm. As the number of committees increases, the utility of the CSA continues to rise, and the gap with the PG-CTS algorithm gradually widens, proving the advantage of CSA in handling large-scale systems. By simulating dynamic events in the IoT environment, the results show that CSA can effectively handle committee joining and leaving events, and quickly restore system stability. CSA aims to optimize the committee scheduling problem in IoT blockchain systems. If applied to the Ethereum mainnet, CSA can address the imbalance in committee scheduling latency and improve the transaction confirmation speed of the Ethereum blockchain. Its core ideas and methods are also applicable to optimization tasks in L2 networks.

## 5. Conclusions

In this paper, we propose an IoT blockchain sharding committee utility optimization scheduling algorithm, called CSA. CSA balances transaction number, cumulative latency, and transaction fees for each epoch, and it addresses these trade-offs using approximation techniques and Markov chain iterative optimization. Theoretical properties of the proposed algorithm, such as convergence time bounds and performance fluctuations caused by committee failures, are critically analyzed. Additionally, a security analysis is performed to evaluate the impact of malicious attacks, such as Sybil and eclipse attacks, on committee failures. Finally, the algorithm’s ability to handle dynamic member committee leaving and joining is also demonstrated.

As blockchain technology continues to evolve and expand into new application domains, future work could explore the scalability of optimization algorithms in large-scale IoT networks and investigate advanced defense mechanisms against emerging attacks. There is also a need to study how to improve the efficiency of the committee selection process in a real-time dynamic environment. The above will be the focus of future research and development to come up with more reliable, secure, and scalable blockchain solutions to meet the demands of IoT. 

## Figures and Tables

**Table 1:** Comparison with existing sharding blockchain schemes.

System	Transaction Type	Utility Maximization	Method	Application Scenarios
RapidChain	UTXO	× *	Splitting Shard	Permissionless Blockchains
OptChain	Data	×	Shard Placement	Permissioned Blockchains with Low Cross-Shard Transactions
SmartChain	Data	×	Adaptive Sharding	IoT Systems with Dynamic Changes
HMMDShard	Account	×	Hidden Markov Model	Dynamic IoT Systems
Aptos	Account	×	Proof of Stake	General-purpose Scalable Blockchain
Sui	Account	×	Narwhal Consensus	Low Latency IoT Applications
Linera	Account	×	Hybrid Consensus	High-Performance IoT and Web3 Applications
Fuel	Account	×	Parallel Execution	High-Performance IoT Networks
SE	UTXO/Account	✓	Markov Chain	Decentralized Blockchain Network
CSA (This Work)	UTXO/Account	✓	Markov Chain	IoT Blockchain Systems Optimization

* The symbol “×” indicates that the scheme does not satisfy the property, while “✓” indicates that the scheme satisfies the property.

## Data Availability

Data used in the experiments were obtained from https://xblock.pro/#/ (accessed on 6 September 2024).
